# Death from colonic disease in epidermolysis bullosa dystrophica

**DOI:** 10.1186/1471-5945-6-2

**Published:** 2006-02-15

**Authors:** Chih-Hsin Hsieh, Che-Jen Huang, Gau-Tyan Lin

**Affiliations:** 1 Department of Orthopaedic Surgery, Chung-Ho Memorial Hospital, Kaohsiung Medical University, No.100, Tz-you 1st road, Kaohsiung 807, R.O.C, Taiwan; 2 Department of Emergency, Chung-Ho Memorial Hospital, Kaohsiung Medical University, No.100, Tz-you 1st road, Kaohsiung 807, R.O.C, Taiwan; 3 Division of Gastroenterology and General Surgery, Chung-Ho Memorial Hospital, Kaohsiung Medical University, No.100, Tz-you 1st road, Kaohsiung 807, R.O.C, Taiwan

## Abstract

**Background:**

Squamous cell carcinomas and renal failure were reported the causes of death in patients with recessive dystrophic epidermolysis bullosa (RDEB). Death from colonic disease in epidermolysis bullosa (EB) is never reported.

**Case presentation:**

We demonstrate a male patient with RDEB. He suffered megacolon due to fecal impaction and died from sigmoid colon perforation with peritonitis at age 35 years.

**Conclusion:**

Constipation is a common clinical feature of RDEB, but fetal complications of chronic constipation are rarely reported. To the author's best knowledge, it has not been reported or recognized in the English literature previously. The aggressive assessment of constipation with fecal impaction is recommended in patients with RDEB.

## Background

Epidermolysis bullosa (EB) encompasses a group of heritable skin disorders manifesting with easy blistering and erosions as a result of minor trauma to the skin. Evaluations of epidemiology of EB in other countries [1-5] were reported. In U.S., the special report of the large size American population for inherited EB was studied[6].

The extracutaneous manifestations are clinically diversified, including respiratory, gastrointestinal, and vesicourinary tract incolvement[6,7]. The dystrophic forms are characterized by tissue cleavage below the lamina densa on the dermal side of the cutaneous basement membrane zone at the level of the anchoring fibrils resulting from mutations in COL7A1, the gene encoding type VII collagen[6-9]. The site and specific nature of the underlying mutation determine the clinical phenotype.

In 2000, Dr. Gau-Tyan Lin had identified a homozygous intronic splice-site mutation at the +1 position of intron 5 (682+1G→A) of COL7A1 in the family of this patient with recessive dystrophic epidermolysis bullosa (RDEB). Consanguinity was noted to be a significant factor of the severe inherited disease[9].

The clinical course of recessive dystrophic epidermolysis bullosa (RDEB) is characterized by generalized skin distribution with early onset at birth, severe blisters, milia, atrophic scarring, dystrophic or absent nails, and repetitive skin infection. The extracutaneous involvements comprise oral ulcers, dental problems, anemia, growth retardation, sparse scalp hair or alopecia, pseudosyndactyly of hands and feet (mitten deformities), gastrointestinal tract manifestations[6,8,9].

Carcinogenesis indicates a major clinical problem for epidermolysis bullosa patients. With increasing age, squamous cell carcinoma (SCC) is a common cause of mortality[7,8]. In addition, renal failure is reported as one of the causes of death among patients with RDEB[10,11]. We demonstrate a patient with RDEB suffered constipation with fecal impaction and eventually demised of sigmoid colon perforation with peritonitis.

## Case presentation

A 35-year-old man sustained Hallopeau-Siemens recessive dystrophic epidermolysis bullosa (RDEB-HS) confirmed by gene analysis. A detailed study of mutation analysis was carried out by Dr. Gau-Tyan Lin[6]. A homozygous intronic splice-site mutation at the +1 position of intron 5 (682+1G→A) in the type VII collagen gene (COL7A1) was identified. His mother was a heterozygous carrier of this mutation[6]. Parental consanguinity was confirmed by the DNA study. With early onset at 7th day postnatal, he suffered blisters and erosions at all skin of his body. He had a characteristic mitten deformity of hands and feet. Since the age of 21 years, he had received lots of surgical procedures including separation of digits, release of flexion contracture, and skin grafting for ulcers, skin defect and pseudosyndactyly of his hands and feet. In the meanwhile, repeated skin infection was experienced. Besides he had anemia, malnutrition, and chronic constipation with stool impaction for years. Laxatives (magnesium oxide 250 mg 3 times per day and sennosides 20 mg before sleep) were given.

**Figure 1:**
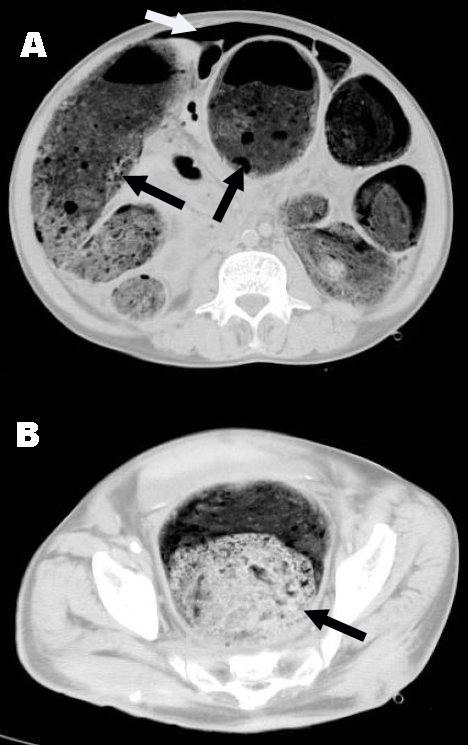
(A) Computed tomography (CT) of abdomen showed extra-luminal free air in the peritoneal cavity (white arrow), and marked distension of colonic loop with fecal impaction (black arrow). (B) Fecal impaction with marked colon distention was noted in the distal sigmoid colon (black arrow).

Due to skin ulcers and wound infection of right hand and right low leg, he was admitted to the hospital. He received wound debridement of right low leg and right hand with separation of first web of right hand. Three days after the operation, abdominal pain and distention were complained. Enema (glycerol 60 ml) was given, but to no avail. Vomiting and abdominal discomfort got worse. White blood count: 25.50 × 1000/ul was noted. Computed tomography (CT) of abdomen demonstrated extra-luminal free air in the peritoneal cavity and stool impaction in the distal sigmoid colon with marked distention of colonic loop (Figure 1). Hallow organ perforation with peritonitis was highly suspected. Emergent laparotomy was performed. During operation, sigmoid colon perforation with ischemic bowel and fecal impaction in the distal sigmoid colon were noted. Hartmann's procedure (resection of ischemic bowel and colostomy ) and intra-abdominal abscess drainage were done. Unfortunately septic shock developed. Intensive management was applied; however, the condition got worse, and he was expired two days after operation.

## Conclusion

Gastrointestinal manifestations of epidermolysis bullosa comprise dysphagia, esophageal stricture, growth failure like esophageal web, constipation and anal lesions[12-15]. They are most common seen in recessive dystrophic epidermolysis bullosa (RDEB), but may also arise in dominant dystrophic epidermolysis bullosa (DDEB), junctional epidermolysis bullosa (JEB) and epidermolysis bullosa simplex (EBS) [6,13,15].

Esophageal involvements are associated with the gradual development of dysphagia. The bullae can be precipitated by the ingestion of food with pain on swallowing. The esophageal web in the upper esophagus or cricopharyngeal area may also result in swallowing difficultily[12-14]. It may lead to low fiber intake and malnutrition. Low fiber intake is likely to contribute to constipation[15].

Anal lesions with bullae, erosions, and scarring may cause painful defecation and result in chronic constipation[13]. Constipation with fecal impaction is a common problem in patients with RDEB[6,12,14,15]. These patients are at the increased risk for developing constipation due to limited oral intake, low fiber intake, anal lesions and excessive loss of fluid through the skin. Chronic constipation may lead to severe bowel distention due to fecal impaction. Large amounts of stool within a redundant colon can be noted on the radiograph of abdomen[13].

For chronic constipation, general standard treatments, such as the use of stool softeners and laxatives including suppositories and enemas, are usually unsuccessful in resolving the problems. Mineral oil is restricted in the treatment of constipation with esophageal obstruction due to the danger of aspiration[13]. Further researches are necessary for drastic prevention and therapy of chronic constipation with fecal impaction in patients with EB.

## Pre-publication history

The pre-publication history for this paper can be accessed here:
